# Short-time effects of spa rehabilitation on pain, mood and quality of life among patients with degenerative or post-surgery musculoskeletal disorders

**DOI:** 10.1007/s00484-022-02381-4

**Published:** 2022-10-08

**Authors:** Maria Chiara Maccarone, Giacomo Magro, Claudio Albertin, Giovanni Barbetta, Salvatore Barone, Camilla Castaldelli, Patrizia Manica, Silvia Marcoli, Magda Mediati, Domenico Minuto, Patrizia Poli, Christian Sigurtà, Gloria Raffaetà, Stefano Masiero

**Affiliations:** 1 Physical Medicine and Rehabilitation School, University of Padova, Padua, Italy; 2 La Residence & Idrokinesis, Abano Terme, Italy; 3 La Contea Terme, Battaglia Terme, Italy; 4 Aquaria Thermal SPA, Terme Di Sirmione, Sirmione, Italy; 5 Thermal Baths of Levico and Vetriolo, Levico Terme, Italy; 6 Bibione Thermae, Bibione, Italy; 7 Second Orthopaedic and Traumatologic Clinic of Pisa, University of Pisa, Pisa, Italy; 8 Rehabilitation Unit, Department of Neuroscience, University of Padova, Padua, Italy

**Keywords:** Rehabilitation, Mud therapy, Balneotherapy, Aquatic therapy, Water-based exercise

## Abstract

Clinical trials have demonstrated traditional spa therapy effects in musculoskeletal disorders (MSDs). This is the first observational study in Italy aimed at evaluating in real-life the short-time effects of spa rehabilitation on pain, mood and quality of life (QoL) among degenerative or post-surgery MSDs patients. Through the involvement of six Italian spa facilities, 160 patients were enrolled; data from 123 patients were finally analysed. Seventy-nine patients (64.3%) accessed the spa for degenerative MSDs, while 44 (35.8%) had a post-surgical condition. All the patients included in the study underwent 12 sessions of water-based exercise (joint exercises, muscle strengthening, gait training, proprioceptive and balance techniques) conducted in thermal or in warm water pools, six sessions per week, for a period of 2 weeks from March 2019 up to October 2019. A group of 45 patients (36.6%) also received traditional thermal therapies, including 12 mud therapy sessions and 12 thermal baths, six times each week, for 2 weeks. Evaluation before and after the treatment included the Numerical Rating Scale (NRS), the Short Form Health Survey (SF-12) and the EuroQol-5D (EQ-5D). The analysis of the scores reported in the questionnaires after the treatment showed a significant improvement in all the scores evaluated. Comparison between patients that performed water-based exercise protocols alone (group A) and patients that in addition to water exercise performed traditional thermal interventions (group B) showed no statistically significant differences in NRSp, NRSa, NRSm, SF-12 PCS, SF-12 MCS and EQ-5D variations; only NRSa value reduction was lower in group B. Sulphate water was found to be associated with a lower reduction of all the scores considered, when compared to the other water types. Patients with degenerative or post-surgery MSDs showed favourable effects on pain, mood and QoL after water exercise training alone or in combination with traditional thermal therapy. Our research provides the first proof that spa rehabilitation can be in real-life conditions an appropriate alternative strategy for post-orthopaedic surgical outcomes recovery. In the future, these results will need to be further investigated.

## Introduction

Musculoskeletal disorders (MSDs) are defined by the World Health Organization as health issues of the locomotor apparatus, including muscles, tendons, bone skeleton, cartilage, ligaments and nerves (Gómez-Galán et al. 2017). Management of MSDs includes non-pharmacological and pharmacological treatments. Among non-pharmacologic treatments, rehabilitative treatments, including aquatic exercise, play a major role. Thermal medicine, one of the most used traditional and complementary interventions for MSDs, can be employed in synergy with water exercise, thanks to the exploitation of a wide range of therapeutic modalities, including thermal baths and mud pack therapy. Traditional spa interventions have been studied extensively in individuals with degenerative MSDs, both in basic science and clinical trials. In these subjects, thermal water effects on symptomatology and inflammation seem to be superior when compared to tap water effects (Fazaa et al. 2014) and to have a longer duration (Özkuk et al. 2018; Fioravanti et al. 2015; Cheleschi et al. 2021). On the other hand, mud therapy can enhance blood flow, connective tissue elasticity (van Tubergen et al. 2001) and plasma levels of β-endorphins, as well as can provide anti-inflammatory effects and regulate the neuroimmunoendocrine system (Altan et al. 2006; Cheleschi et al. 2021).

Recently, the spa setting is being increasingly employed to conduct specific rehabilitative activities aimed at patients with MSDs. This setting enables to not overload joints, especially in older or obese subjects (Yurtkuran et al. 2006; Fioravanti et al. 2010), while reducing pain perception thanks to the combination of mechanical (buoyancy, viscosity, etc.), thermal and chemical water properties. Aquatic therapy in thermal water has also been proposed for post-orthopaedic surgery patients, demonstrating positive effects (Musumeci et al. 2018; Masiero et al. 2020a).

Even if many clinical trials have investigated the impact of traditional BT on MSDs, few studies have evaluated the role of real-life spa rehabilitative protocols, combining traditional spa treatments with specific water-based exercise. A previous observational, longitudinal, questionnaire-based study (the Naiade project) considered patients suffering from rheumatic, respiratory, dermatologic, gynaecologic, otorhinologic, urinary, vascular and gastroenteric disorders admitted to 297 of the 340 certified Italian spa centres (Fioravanti et al. 2003). Nevertheless, this is the first observational study in Italy aimed at investigating the short-term effects of a real-life spa rehabilitative protocol (aquatic therapy with or without the addition of traditional thermal therapy) on pain perception, mood and QoL among degenerative and post-orthopaedic surgery MSDs patients.

## Materials and methods

### Study design

We conducted an observational study in six Italian spa facilities, located in the northeast and central Italy (in the regions of Veneto, Lombardy, Tuscany and Trentino Alto Adige), reporting comparisons between the results of two questionnaire-based inquiries: an “entry” inquiry, before the treatment, and a second inquiry after the treatment.

### Participants

The study population was recruited among the users of the spa facilities who have a medical prescription for a spa treatment. The subjects underwent spa treatments from March 2019 up to October 2019.

Main inclusion criteria comprise subjects of both sex over the age of 18, diagnosis of degenerative MSDs (knee or hip OA, low back pain, tendinopathy, etc.) or post-surgical musculoskeletal concerns (knee or hip prosthetic replacement). Enrolled subjects should be capable of giving their informed consent. The study was conducted in accordance with the principles of the Declaration of Helsinki.

Patients underwent a thorough clinical assessment, and when necessary, appropriate laboratory and/or instrumental investigations, in order to confirm diagnosis of the referring physician, exclude contraindications to thermal therapy and establish the need for traditional spa treatment in addition to water exercise, according to common spa treatment prescription guidelines.

Exclusion criteria include contraindications to thermal water treatment (i.e. skin ulcers, severe burns, heart failure, respiratory failure, urinary or faecal incontinence), impaired cognitive functions (mini mental status examination < 24), epilepsy, pregnancy, oncological or psychiatric comorbidities, inability to properly comprehend and sign informed consent.

### Intervention and procedure

The study was carried out in different thermal settings (Sirmione, Lombardy region, Italy; Abano Terme, Veneto region, Italy; Battaglia Terme, Veneto region, Italy; Levico Terme, Trentino Alto Adige region, Italy; Bibione, Veneto region, Italy; Casciana Terme, Tuscany region, Italy), each one with peculiar characteristics in waters’ mineral content and temperature.

Sirmione’s hyperthermal water (temperature at source 69 °C) is classified as sulphurous water, based on its chemical-physical characteristics. Thermal waters of the Euganean Thermal Basin, comprising Abano Terme and Battaglia Terme, are classified as salso-bromo-iodic waters. Abano Terme’s thermal water springs at a temperature of around 80 °C, while Battaglia Terme’s water springs at a temperature of 87 °C. Levico Terme’s mineral-rich water has a high content of sulphate and iron (sulphurous-arsenical-ferruginous water, temperature at source 9.7 °C). Bibione’s mineral-rich water is classified as alkaline-sodium bicarbonate-fluoride water and it springs at 52 °C. Casciana Terme’s water is a bicarbonate-sulphate-calcic water and flows from the spring at a constant temperature of 35.7 °C.

Rehabilitative interventions in the different spa facilities were prescribed by a physical medicine and rehabilitation physician (PMR). The rehabilitation treatment consisted of twelve sessions of water-based exercise (joint exercises, muscle strengthening, gait training, proprioceptive and balance techniques) conducted in thermal or warm water (Levico and Vetriolo) pools, six sessions per week, for a period of 2 weeks. A group of 44 patients also received traditional thermal therapies, including 12 mud therapy sessions and 12 thermal baths, six times each week, for 2 weeks. Rehabilitation treatments, as well as traditional spa interventions, have been standardised through education sessions conducted by the University of Padua personnel before starting the study and addressed to the staff of the various spa facilities. In addition, our research team trained and supervised each spa employee also in patient enrolment, anamnestic collection and administration of questionnaires.

The assessments were performed immediately before starting the treatment (t0) and at the end of the treatment (t1). Originally, follow-up assessments were scheduled 1 and 3 months after the end of the spa interventions; unfortunately, the emergency situation due to COVID-19 pandemic severely limited the final stage of our study and we decided to not take into consideration the data collected at the follow-ups as they were affected by external events.

During the entire treatments, any adverse events or incidents were recorded.

### Outcome measures

Three different evaluation scales in the Italian validated version were employed:

- The Numerical Rating Scale (NRSp): a quantitative one-dimensional numerical scale for pain assessment. Its compilation requires the patient to select the number, from 0 to 10, that best describes the intensity of his pain at that moment. Anxiety and mood were also assessed with the same scale (NRSa and NRSm, respectively, from 0 to 10).
- The 12-Item Short Form Health Survey (SF-12): a questionnaire readjusted from a larger version, the 36-Item Short Form Health Survey (SF-36), used to investigate the perception of personal psychophysical conditions, frequently employed in the rehabilitation field. The SF-12 results, dual and expressed by the acronyms PCS (Physical Component Summary, from 0 to 100) and MCS (Mental Component Summary, from 0 to 100), can adequately summarise the size of the patient’s impairment both from a physical and mental point of view (Sampogna et al. 2019). A score of 50 or less on the PCS has been recommended as a cut-off to determine a physical condition, while a score of 42 or less on the MCS may be indicative of clinical depression (Ware et al. 1995).
- The EuroQol-5D (EQ-5D): a standardised and non-disease-specific tool for describing and assessing a person’s health status. It is a particularly simple, easy-to-use questionnaire. The scale assesses QoL using a 5-point scale that includes mobility, self-care, regular activities, pain/discomfort and anxiety/depression. EQ-5D health state can be summarised by a single summary number (index value), which reflects how good or bad a health state is. Health state index scores generally range from less than 0 (where 0 is the value of a health as bad as being dead; negative values representing values as worse than dead) to 1 (the value of full health).

In addition, a questionnaire designed by our research team to collect anamnestic data was submitted to the patients. Information such as age, sex, type of pathology, BMI, pain characteristics (intensity, duration, locations) and the occurrence of anxiety or mood alterations were collected and gathered.

### Statistical method

Data were analysed with R 4.1.2. The *t*-test function of the stats library was employed to determine if there was a significant difference between the means of two groups following a Student’s *t*-distribution under the null hypothesis. Student’s one-sample *t*-test was employed for quantitative variables, in particular to compare the scores obtained in the various indicators before and after treatments. Welch’s two-sample *t*-test was employed to compare the results obtained in different subgroups, where it was necessary to compare a maximum of two categories. The ANOVA function of the same package was used to compare results in more than two categories. The level of statistical significance was set at 95%; *p* value significance was defined for values ≤ 0.05.

## Results

### Patients’ demographic and anamnestic characteristics

**Fig. 1:**
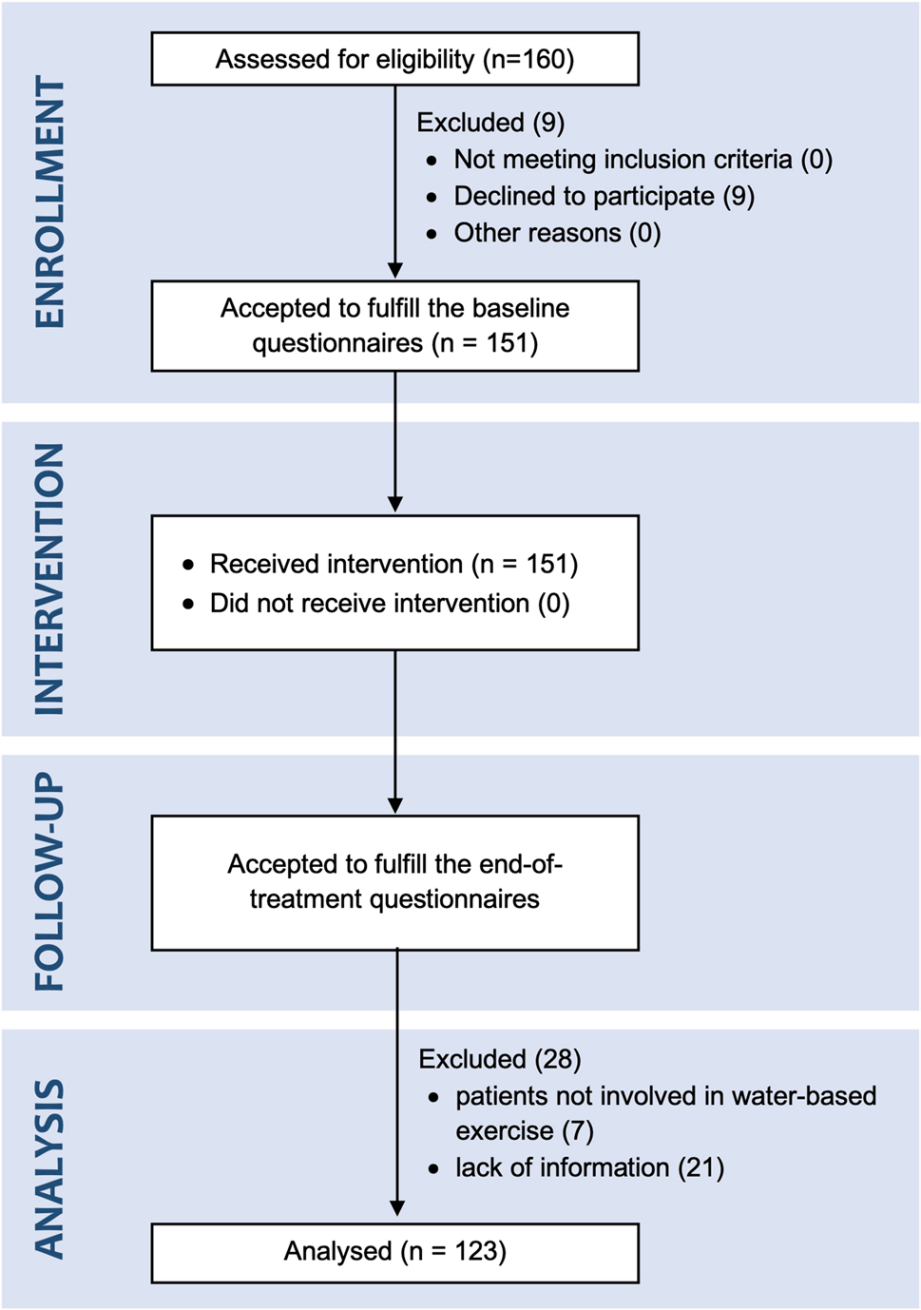
Patients’ selection process

A total of 160 patients were initially assessed for eligibility. Of these, 9 patients did not consent to participate in the study. Hence, 151 patients were included in the study; all underwent 12 sessions of water exercise in the thermal environment, completed the rehabilitation sessions and filled in the questionnaires at the end of the treatment. Of those who agreed to participate in the longitudinal study, 21 were excluded from the analysis of data because they did not adequately complete the questionnaires administered. Seven patients were excluded from the data analysis because they did not perform water-based exercise. The patients’ selection process is illustrated in Fig. 1.

The demographic variables of the population and the types of interventions performed are described in Table 1.

**Table 1:** Patients’ demographic variables and intervention characteristics. *BMI*, body mass index

Demographic variables	Sex	Male	38 (30.9%)
		Female	85 (69.1%)
	Age (mean 64.69 years)	< 65 years	50 (40.7%)
		≥ 65 years	73 (59.3%)
	BMI (mean 25.55)	Healthy (BMI 18.5–24.9)	56 (45.5%)
		Overweight (BMI 25.0–29.9)	53 (43.0%)
		Obese (BMI ≥ 30.0)	14 (11.4%)
	Medical history	Degenerative MSDs	79 (64.3%)
		Post-surgical conditions	44 (35.8%)
Intervention	Water-based exercise		123 (100%)
	Traditional spa treatments in addition		45 (36.6%)
	Water type	Sulphurous water	31 (25.2%)
		Sulphate water	74 (60.2%)
		Salso-bromo-iodic water	9 (7.3%)
		Alkaline-bicarbonate-sodium-fluoride water	9 (7.3%)

### Outcome evaluation

Considered as a whole, the spa rehabilitative treatments (including both water-based exercise in thermal water and traditional spa interventions) had a statistically significant effect on patients’ symptomatology and clinical condition. In the entire population, there was a statistically significant reduction in the NRSp scale values (*p* < 0.001) and in the NRSa scale (*p* < 0.001). A statistically significant increase was recorded in the SF-12 PCS (*p* < 0.001), in the SF-12 MCS (*p* < 0.001), in the EQ-5D index (*p* < 0.001) and in the NRSm scale (*p* = 0.005) (Table 2). No undesirable effects leading to discontinuation or suspension of treatments have been reported.

**Table 2:** Score variation (t1-t0) before and after treatment referring to the total of patients, 95% confidence interval (CI) using the Student’s one-sample *t*-test (H1: score variation mean differs from zero). *SD*, standard deviation; *NRSp*, Numerical Rating Scale assessing pain; *NRSa*, Numerical Rating Scale assessing anxiety; *NRSm*, Numerical Rating Scale assessing mood; *SF-12*, 12-Item Short Form Health Survey; *PCS*, Physical Component Summary; *MCS*, Mental Component Summary; *EQ-5D*, EuroQol-5D

Scores	t0	t1	t1-t0			
	Mean	Mean	Mean	SD	CI(0.95)	*p* value
NRSp variation	5.25	2.76	− 2.47	0.14	(− 2.75; − 2.19)	< 0.001
NRSa variation	3.64	1.95	− 1.76	0.19	(− 2.14; − 1.38)	< 0.001
NRSm variation	5.25	6.05	0.75	0.26	(0.23; 1.27)	0.005
SF-12 PCS variation	36.12	40.14	4.03	0.67	(2.71; 5.36)	< 0.001
SF-12 MCS variation	45.02	49.92	5.11	0.90	(3.32; 6.9)	< 0.001
EQ-5D variation	0.41	0.66	0.26	0.27	(0.20; 0.31)	< 0.001

Comparison between patients that performed water-based exercise protocols alone (group A) and patients that in addition to water exercise performed traditional spa interventions (group B, only composed by patients with degenerative MSDs) showed no statistically significant differences in NRSp, NRSm, SF-12 PCS, SF-12 MCS and EQ-5D score changes. The comparison between the two groups shows only a significant difference in NRSa values (Table 3).

**Table 3:** Score variation (t1-t0) before and after treatment referring to group A and group B using the Welch’s two-sample *t*-test (H1: score variation mean differs from zero). *SD*, standard deviation; *NRSp*, Numerical Rating Scale assessing pain; *NRSa*, Numerical Rating Scale assessing anxiety; *NRSm*, Numerical Rating Scale assessing mood; *SF-12*, 12-Item Short Form Health Survey; *PCS*, Physical Component Summary; *MCS*, Mental Component Summary; *EQ-5D*, EuroQol-5D

Scores	Group A			Group B			Group A (t1-t0) vs group B (t1-t0)		
	t0	t1	t1-t0	t0	t1	t1-t0	Mean	SD	*p* value
	Mean	Mean	Mean	Mean	Mean	Mean			
NRSp variation	4.88	2.38	− 2.5	5.76	3.34	− 2.42	− 0.08	0.29	0.79
NRSa variation	4.18	2.02	− 2.16	3.02	1.98	− 1.04	− 1.12	0.35	0.001
NRSm variation	5.06	6.07	1.01	5.69	5.98	0.29	0.72	0.59	0.22
SF-12 PCS variation	34.70	38.61	3.91	38.58	42.83	4.24	− 0.33	1.43	0.82
SF-12 MCS variation	43.20	49.52	6.32	47.12	50.13	3.01	3.31	1.82	0.07
EQ-5D variation	0.36	0.66	0.30	0.50	0.68	0.18	0.12	0.06	0.06

**Fig. 2:**
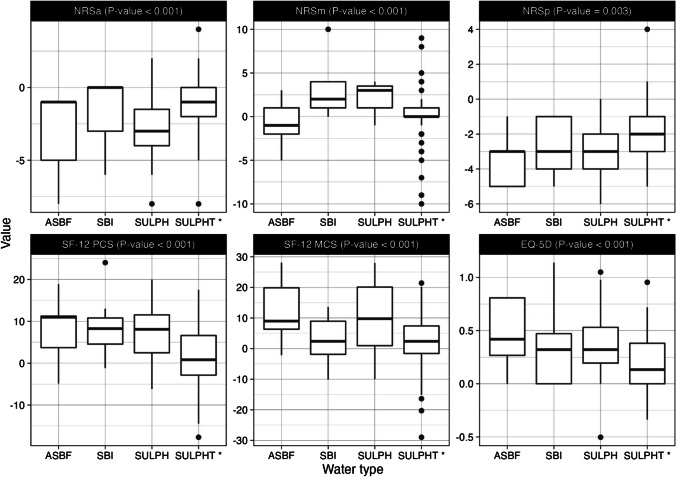
Graphical representation of the score variation (t1-t0) in four subgroups of patients distinguished by the type of water in which treatment was conducted. *p* value calculated using ANOVA tests. NRSa, Numerical Rating Scale assessing anxiety; NRSm, Numerical Rating Scale assessing mood; NRSp, Numerical Rating Scale assessing pain; SF-12, 12-Item Short Form Health Survey; PCS, Physical Component Summary; MCS, Mental Component Summary; EQ-5D, EuroQol-5D; SULPH, sulphurous; ASBF, alkaline-sodium bicarbonate-fluoride; SBI, salso-bromo-iodic; SULPHT, bicarbonate-sulphate-calcic

Comparison of the treatments conducted in the different water types using ANOVA tests showed statistically significant differences. In particular, sulphate water was found to be associated with a lower reduction of all the scores considered, when compared to the other water types (Fig. 2).

## Discussion

To our knowledge, this is the first observational study in Italy to evaluate under real clinical conditions and in different spa facilities the short-time effects on mood, pain perception and QoL of spa rehabilitation among subjects with post-surgery or degenerative MSDs. Patients attending six Italian spa facilities and undergoing aquatic therapy and, if necessary, traditional spa treatments were evaluated. At the end of the treatment, a statistically effectiveness of balneotherapy interventions in the short term, and after short rehabilitative interventions, in the scales used to assess QoL, mood and pain perception, was shown, in line with the existing literature. Our research provides the first proof that spa rehabilitation can be in real-life conditions an appropriate alternative strategy for post-orthopaedic surgical outcomes recovery.

The thermal environment has been demonstrated to be favourable for rehabilitative interventions in post-orthopaedic surgery conditions (Liebs et al. 2012; Paoloni et al. 2017) and degenerative MSDs (Kamioka et al. 2010; Bartels et al. 2016). BT modalities can contribute to pain relief thanks to the reduction of muscle spasm, to the modulation of pro-inflammatory molecules production and to the increased pain threshold due to mineral-rich water pressure, temperature and content (Sukenik et al. 1990; Ardiç et al. 2007; Ciprian et al. 2013; Fraioli et al. 2013; Bağdatlı et al. 2015; Bazzichi et al. 2013; Lim et al. 2010; Masiero et al. 2018). Mud therapy, on the other hand, a natural treatment combining a solid component with thermal water enhances blood flow, increases connective tissue elasticity (van Tubergen et al. 2001) and ameliorates plasma levels of β-endorphins, corticotropin, cortisol and prolactin secretion, resulting in reduced pain perception (Altan et al. 2006). Minimising the risk of joint injuries, thermal environment can be proposed as a safe and effective rehabilitative setting even for older patients suffering from degenerative MSDs (Karagülle et al. 2016; Masiero et al. 2020a; Maccarone et al. 2021a). In clinical trials, water-based exercise in thermal water has been shown to be helpful in the recovery of patients with post-surgical MSDs; however, there is still poor evidence from observational studies. Aquatic exercises performed 5 days a week for 2 weeks in a 33 °C thermal pool revealed better pain and QoL changes than a non-aquatic exercise protocol in postoperative chronic low back pain patients (Yolgösteren et al. 2021). Daily sessions for 2 weeks of land-based and aquatic therapy in thermal pool showed an improvement in motor and functional recovery and a positive impact on QoL in patients after hip arthroplasty (Musumeci et al. 2018).

From the analysis of our data, patients who performed traditional thermal treatments in addition to water-based exercise seemed to present a lower reduction in anxiety. This finding must be read in the light of the fact that patients who performed traditional thermal treatments had degenerative, chronic MSDs. Chronic MSDs have been shown to be linked to mood and anxiety disorders, depressive conditions and low QoL (Heikkinen et al. 2019). In literature, it has been shown that in MSDs with a psychological component, such as fibromyalgia, spa interventions could positively influence anxiety and mood states (Branco et al. 2016; Fraioli et al. 2013; Maccarone et al. 2020), probably through an increase in cortisol levels, thanks to a rise in ACTH production (Antonelli et al. 2018, Latorre-Román et al. 2015).

Previously, improvements > 3.77 in MCS and > 3.29 in PCS have been considered minimal clinical important differences (MCIDs) in patients with subacute and chronic low back pain (Díaz-Arribas et al. 2017). Our questionnaires showed variations above the MCID previously described in group A, while in group B SF-12 MCS variation at the end of the treatment was less than 3.29. However, these MCIDs should be considered only as an indication since they do not refer to the specific MSDs we considered.

Finally, in our study, waters’ mineral content appears to play a specific role in patients’ response in terms of QoL, mood and pain perception. Interventions in sulphate waters demonstrated less improvements than interventions conducted in other water types. However, this result must be interpreted with caution because the number of patients treated with the various types of water varied, making generalisations problematic. Indeed, even if sodium-sulphate and sulphate-bicarbonate mineral waters are widely used for hydroponic therapy of biliary and digestive diseases (Mennuni et al. 2014), it is well documented in the literature how mud maturated with sodium-chloride sulphate mineral water and sulphate BT exert an anti-inflammatory and pain relief action in patients suffering from OA (Costantino et al. 2020). Sulphate-water patients conducted only the traditional thermal interventions in thermal pools, whereas the exercise protocol was conducted in warm water. This element certainly affected the results obtained, since only part of the treatment was carried out in thermal water, further confirming the positive role of thermal water on psychological concerns. Indeed, the positive effects on QoL and mood after rehabilitative interventions in the thermal environment should derive not only from the chemical and physical properties of the waters (Kesiktas et al. 2012; Onat et al. 2014; Maccarone et al. 2021b; Scanu et al. 2021; Cheleschi et al. 2021) but also from the beneficial psychological role played by the environment itself (Maccarone et al. 2020; Masiero et al. 2020a).

## Study limitations

The tiny sample size is undeniably a weakness of this study. However, because of the concurrent pandemic COVID-19, access to spa facilities was limited due to travel restrictions and reductions in non-urgent treatments. The lack of a control group is also a limitation of our study. Moreover, there was no assessment of patients’ mobility impairment and, despite efforts to ensure that the same type of treatment was provided in each centre (educational sessions for the staff of the various spa facilities conducted by researchers from the University of Padua), this may have differed.

The pandemic’s lockdown phase imposed significant limits; indeed, the 1- and 3-month follow-ups scheduled following the end of therapy were not conducted. As a result, the data gathered can only be considered partial, although providing crucial information about the effects of rehabilitative therapy in the Italian thermal environments.

Finally, this study focuses solely on MSDs. In the future, additional disorders may be examined.

## Conclusions

The favourable effects of spa rehabilitation on pain, mood and QoL for patients with post-surgery or degenerative MSDs seem to be suggested by this study. The spa environment could increasingly be a resource for post-surgical patients, providing an out-of-hospital alternative for rehabilitation. The addition of traditional thermal treatments should be a viable strategy for those suffering from degenerative MSDs.

Continuing with the planned follow-up evaluations and future studies will allow evaluation of long-term effects and further investigation of the role of water type in clinical effects.
